# Covalent modification of a ten-residue cationic antimicrobial peptide with levofloxacin

**DOI:** 10.3389/fchem.2014.00071

**Published:** 2014-09-18

**Authors:** Carlos A. Rodriguez, Emilios A. Papanastasiou, Melanie Juba, Barney Bishop

**Affiliations:** Department of Chemistry and Biochemistry, George Mason UniversityFairfax, VA, USA

**Keywords:** cationic antimicrobial peptide, peptide conjugate, antibiotics, peptide modification, levofloxacin

## Abstract

The rampant spread of antibiotic resistant bacteria has spurred interest in alternative strategies for developing next-generation antibacterial therapies. As such, there has been growing interest in cationic antimicrobial peptides (CAMPs) and their therapeutic applications. Modification of CAMPs via conjugation to auxiliary compounds, including small molecule drugs, is a new approach to developing effective, broad-spectrum antibacterial agents with novel physicochemical properties and versatile antibacterial mechanisms. Here, we've explored design parameters for engineering CAMPs conjugated to small molecules with favorable physicochemical and antibacterial properties by covalently affixing a fluoroquinolone antibiotic, levofloxacin, to the ten-residue CAMP Pep-4. Relative to the unmodified Pep-4, the conjugate was found to demonstrate substantially increased antibacterial potency under high salt concentrations. Historically, it has been observed that most CAMPs lose antibacterial effectiveness in such high ionic strength environments, a fact that has presented a challenge to their development as therapeutics. Physicochemical studies revealed that P4LC was more hydrophobic than Pep-4, while mechanistic findings indicated that the conjugate was more effective at disrupting bacterial membrane integrity. Although the inherent antibacterial effect of the incorporated levofloxacin molecules did not appear to be substantially realized in this conjugate, these findings nevertheless suggest that covalent attachment of small molecule antibiotics with favorable physicochemical properties to CAMPs could be a promising strategy for enhancing peptide performance and overall therapeutic potential. These results have broader applicability to the development of future CAMP-antibiotic conjugates for potential therapeutic applications.

## Introduction

The emergence and spread of bacterial resistance to conventional antibiotics has resulted in interest in cationic antimicrobial peptides (CAMPs) as a potential therapeutic alternative (Gordon et al., 2005; Seo et al., 2012). These peptides demonstrate broad-spectrum antimicrobial activity and are essential elements of innate immunity in higher organisms. Yet, despite their extensive use in nature, bacteria have failed to develop wide-spread resistance to CAMPs, which has not been the case for conventional antibiotics (Zasloff, 2002). While CAMPs demonstrate extensive sequence and structural diversity, they are generally rich in cationic amino acids and adopt amphipathic structures that result in spatial partitioning of basic and hydrophobic residues. These peptides are thought to exert their antibacterial effect *via* mechanisms that at some level involve the targeting of anionic bacterial membranes through non-specific electrostatic and hydrophobic interactions. Many CAMPs are thought to also bind bacterial receptors and enzymes, or otherwise interfere with essential cellular processes (Epand and Vogel, 1999; Brogden, 2005; Jenssen et al., 2006).

While CAMPs represent a promising class of prospective therapeutics, their development as viable treatments has proven challenging, with one major factor being that the effectiveness of many CAMPs are diminished or even abolished under salt concentrations consistent with physiologically relevant environments (Park et al., 2004; Yu et al., 2011; Chu et al., 2013). Researchers have investigated various strategies, including conjugation to auxiliary compounds, in order to enhance CAMP antibacterial performance and potential therapeutic utility (Arnusch et al., 2012; Devocelle, 2012). Conjugation is an appealing strategy because it provides a means for introducing new functionalities and directly enhancing CAMP antibacterial activity. For example, CAMPs have been conjugated to fatty acids (Li et al., 2013), sugars (Pal et al., 2011), pheromones, antibodies, synthetic polymers, and small molecule drugs to yield peptide constructs with improved antibacterial activity relative to their respective unmodified parent peptide variants (Devocelle, 2012). However, while conjugation can improve the performance of CAMPs, it has also been shown in some instances to abrogate antibacterial effectiveness (Chu-Kung et al., 2004; Radzishevsky et al., 2005). Therefore, the effect that conjugation may have on CAMP structure and physicochemical properties (e.g., charge, hydropathy, and sterics, etc.) must be considered when engineering constructs. Conjugation of CAMPs to small molecules with favorable physicochemical properties (such as hydrophobic and/or cationic functionalities) would provide a highly controlled means for augmenting peptide antibacterial performance. The incorporation of small molecule antibiotics in CAMP-based conjugates presents a particularly intriguing strategy, as these compounds could influence peptide effectiveness not only *via* alteration of physicochemical properties, but also through their intrinsic antibacterial activities. In this regard, fluoroquinolone antibiotics, which present both hydrophobic (e.g., aromatic and aliphatic moieties) and cationic functionalities, represent attractive candidates. Additionally, from a synthetic perspective, the carboxylate moieities that fluoroquinolones contain lend themselves to ready conjugation with CAMP primary amino groups.

In our previous studies, we found that Pep-4, a highly cationic peptide with a sequence (RGRRSSRRKK-NH_2_) based on the C-terminal portion of human beta defensin-3 (hBD-3), retained significant antibacterial activity following acetylation of its N-terminal and side chain amino groups, despite the associated reduction in charge from +8 to +5 (Papanastasiou et al., 2009). These results suggest that this peptide is amenable to chemical modification and may be a good candidate for incorporation in CAMP-conjugates. Additionally, the sequence of Pep-4 provides a limited number of reactive functionalities, allowing convenient chemical modification with small molecules while reducing the potential for deleterious side reactions and byproducts. These qualities also facilitate isolation and characterization. Moreover, the small size of Pep-4 makes it especially appealing for use in CAMP-conjugates, as it lends itself to cost effective synthesis (Seo et al., 2012).

In order to explore design parameters for engineering antibacterial CAMP-conjugates and the incorporation of small molecules, such as antibiotics, we have synthesized a CAMP-conjugate consisting of a fluoroquinolone, levofloxacin (LVFX), covalently attached to the scaffold peptide Pep-4 *via* the primary amino groups present in the peptide. These studies have focused on evaluating the antibacterial performance of the Pep-4-LVFX Conjugate, P4LC, against the model gram-negative bacterium *E. coli* and gram-positive bacterium *B. cereus*. Our studies reveal that incorporation of LVFX significantly enhances the effectiveness of the conjugate under physiologically relevant environmental salt concentrations relative to the unmodified scaffold peptide Pep-4. These findings suggest that conjugation of CAMPs to auxiliary compounds, such as small molecule antibiotics with favorable physicochemical properties, to generate hybrid constructs could be a promising strategy for enhancing peptide therapeutic potential.

## Materials and methods

Levofloxacin (LVFX), N-methylmorpholine (NMM) and resazurin were purchased from Sigma-Aldrich, Co. LLC (St. Louis, MO, USA). Bicinchoninic acid (BCA) and SYTOX Green were purchased from ThermoFisher Scientific, Inc. (Waltham, MA, USA). Fluoro-N,N,N′,N′-tetramethylformamidinium hexafluorophosphate (TFFH) was purchased from EMD Millipore (a division of Merck KGaA; Darmstadt, Germany), Fmoc-Gly-Wang resin from Peptides International, Inc. (Louisville, KY, USA), and DiSC(3)5 from Anaspec, Inc. (Fremont, CA, USA). Deionized water (dH_2_O) was prepared using a Milli-Q Synthesis A10 system (Millipore). The bacterial strains *E. coli* (#25922) and *B. cereus* (#11778) were purchased from American Type Culture Collection (ATCC, Manassas, VA, USA). Pep-4 (≥95% purity) was custom synthesized by Genscript USA, Inc. (Piscataway, NJ, USA). Melittin (≥95%) was purchased from Anaspec, Inc., and indolicidin was synthesized (≥95% purity) by AAPPTec, LLC (Louisville, KY, USA). Peptide identities were verified *via* matrix assisted laser desorption ionization time-of-flight (MALDI-TOF) mass spectrometry using a Shimadzu AXIMA Performance. Additionally, Pep-4 and the conjugate were submitted for amino acid analysis (UC Davis Genome Center Proteomics Core Facility) in order to establish molar concentrations.

### P4LC synthesis and purification

The carboxyl group of LVFX allowed for conjugation to Pep-4 *via* direct acylation of the peptide's N-terminal and lysine side chain amino groups. LVFX was preactivated by converting its carboxyl moiety into an acyl fluoride in order to facilitate nucleophilic attack by peptide amino groups. Equimolar amounts of LVFX (6.7 mg, 18.6 mmol) and TFFH (4.90 mg, 18.6 mmol) were dissolved in 500 μL of anhydrous DMF, and 20 μL of NMM was then added to the solution. This reaction was allowed to stir at room temperature (rt), under N_2_ in the dark. After 1 h, Pep-4 (2.4 mg, 1.86 mmol) dissolved in 200 μL of anhydrous DMF was added to the reaction and allowed to continue stirring at rt, under N_2_ in the dark. While the coupling reaction was stirring, Fmoc-Glycine-Wang resin (200 mg, 0.63 mmol/g amine loading) was suspended in 8 mL of DMF with agitation for 30 min to swell the resin, and the solvent was then removed *via* vacuum filtration. The suspension and filtration process was repeated. The swelled resin was then suspended in 8 mL of 50% piperidine in DMF for 30 min in order to remove the Fmoc protecting group. The reaction mixture was vacuum filtered and the resin resuspended in 8 mL of DMF and agitated for 15 min. The resin suspension was then vacuum filtered, and the deprotected particles were transferred to the reaction vessel. Following addition of the resin, the reaction was left stirring in the dark under N_2_, overnight. Thereafter, the contents of the reaction vessel were filtered through a course frit directly into cold diethyl ether, and the resulting suspension was centrifuged so as to pellet the precipitated material. The pelleted precipitate was then dried under vacuum in order to remove residual ether. Crude conjugate was subsequently dissolved in 10 mL of 10 mM NaCl and dialyzed (0.5–1 kD M_w_ cutoff) in 10 mL 10 mM NaCl against 2 L dH_2_O, which was replaced every 8 h (3x). The dialyzed solution was then lyophilized to yield 3.1 mg of purified P4LC (~87% yield).

### Mass spectrometry

Samples dissolved in dH_2_O (1 pmol/μL) were mixed with α-cyano-4-hydroxycinnamic acid (CHCA) matrix solution (10 mg/ml in 50:50 water/acetonitrile with 0.1% TFA) and deposited onto a stainless steel target plate (Shimadzu Kratos Analytical) in 1 μL aliquots. Mass spectra were collected using a Shimadzu AXIMA Performance MALDI-TOF mass spectrometer equipped with a 337 nm N_2_ laser in reflectron mode and represent the average of at least 50 accumulated profiles. The laser repetition rate was set to 50 Hz, and laser power was set to between 50 and 70%. The instrument was externally calibrated using angiotensin II, angiotensin I, and Adrenocorticotropin hormone (fragment 18–39). Lower molecular weight regions of spectra were assessed with the ion gate turned off, and control spectra were generated for matrix alone. CHCA peaks were used for internal calibration of the lower molecular weight regions of sample spectra. Matrix m/z values were verified using MassBank Mass Spectral Database (Accession: MCH00005).

### Antibacterial performance assays

The antibacterial activities of P4LC, Pep-4, LVFX, and free Pep-4 in combination with free LVFX (Pep-4/LVFX) were determined in a microplate-based assay using resazurin as an indicator for cell viability. Metabolically active cells convert resazurin, which is not fluorescent, to the highly fluorescent resorufin (530/590 nm_em_), and the rate of this conversion provides a means for quantifying the concentration of viable bacteria (Shiloh et al., 1997; Lemos and Carareto-Alves, 1998; Okuda et al., 2006; Mariscal et al., 2009). In order to assess the effect environmental ionic strength had on antibacterial effectiveness, assays were conducted in buffers with the following ionic strengths: 26 mM, 111 mM, and 167 mM.

Frozen enumerated aliquots of bacteria were thawed and diluted into sterile buffer to a bacterial concentration of 2 × 10^6^ CFU/mL. Wells of a 96-well polypropylene microtiter plate (Greiner Bio-One) were charged with 50 μL aliquots of buffer solutions containing serially diluted peptide, conjugate, or drug followed by 50 μL aliquots of diluted bacterial cells. Assays for each compound were prepared in triplicate. Control wells were prepared where the peptide/conjugate/drug solution was replaced with an equal volume of buffer alone. Following addition of bacteria, the microtiter plates were incubated for 3 h at either 37°C (*E. coli*) or 30°C (*B. cereus*). Aliquots of PBS solution (100 μL) containing resazurin and MHB were then added to each well to afford final concentrations of 100 μM resazurin and 0.2% MHB (wt/vol) for *E. coli* and 12.5 μM resazurin and 0.05% MHB (wt/vol) for *B. cereus*. Plates were then immediately placed in a SpectraMax Gemini EM plate-reading fluorometer for overnight monitoring of fluorescence (530 nm_ex_/590 nm_em_) at either 37°C (*E. coli*) or 30°C (*B. cereus*) with intermittent shaking. Onset time of half maximal fluorescence (T_0.5_) observed for each well was computed using the microplate data analysis software. In order to determine bacterial survival, it was necessary to establish correlations between bacterial concentration (CFU/mL) and T_0.5_ values. Therefore, standard curve equations were generated in preliminary experiments using bacterial suspensions serially diluted (~10^6^ CFU/mL—10^3^ CFU/mL) in each of the three buffers used in the antibacterial assays. Plotting initial bacterial concentrations (determined *via* dilution plating onto MHB Agar plates) against observed T_0.5_ values afforded equations 1 (*E. coli*) and 2 (*B. cereus*), which were used to interpolate viable bacterial concentration (CFU/mL) following incubation with peptide, conjugate or drug. The Y-intercept (Y_int_) values for equations generated for the varied ionic strength buffer conditions were 9.41 (26 mM), 9.19 (111 mM), and 9.24 (167 mM) for *E. coli*, and 5.91 (26 mM), 6.20 (111 mM), and 6.36 (167 mM) for *B. cereus*.

(1) $$\log({\text{CFU/mL}}_{E.coli})=-2.00\;\times \;10^{-4}({\text{T}}_{0.5})+{\text{Y}}_{\text{int}}$$

(2) $$\log({\text{CFU/mL}}_{B.cereus})=-1.00\;\times \;10^{-4}({\text{T}}_{0.5})+{\text{Y}}_{\text{int}}$$

Antibacterial effectiveness against each microbe was determined by plotting bacterial survival as a function of the log of the concentration of P4LC, Pep-4, LVFX, or Pep-4/LVFX, and fitting the resulting data to a variable-slope sigmoidal regression model (equation 3) using Graphpad Prism 5 (GraphPad Software, Inc.). In this equation, log(EC_50_) represents the log of the concentration of P4LC, Pep-4, LVFX, or Pep-4/LVFX required to kill half of the bacterial population, where S_min_ and S_max_ correspond to the minimal and maximal bacterial survival values (respectively), and HS is the parameter defining the steepness of the transition slopes of sigmoidal survival curves.

(3) $$\begin{array}{l} \text{Bacterial Survival}={\text{S}}_{\text{min}}+({\text{S}}_{\text{max}}-{\text{S}}_{\text{min}})/\\ \;(1+10^{\text{log}(\text{EC50})-\text{log}(\text{PC})*\text{HS}}) \end{array}$$

Best-fit log (EC_50_) values generated for each compound were used to compare the antibacterial effectiveness of P4LC, Pep-4, LVFX, and Pep-4/LVFX against each bacterium. Antilogs of the log (EC_50_) values, the EC_50_ values, are tabulated in Table 1 along with their respective 95% confidence intervals.

**Table 1:** **Antibacterial effectiveness**.

	**P4LC**	**Pep-4**	**Pep-4/LVFX**	**LVFX**
***E. COLI***				
μ_1_	0.0167 (0.0132–0.0230)	0.0168 (0.0123–0.0234)	0.00375 (0.00314–0.00447)	0.00281 (0.00228–0.00344)
μ_2_	1.71 (1.60–1.81)	28.3 (17.2–46.7)	0.00487 (0.00387–0.00617)	0.00223 (0.00164–0.00303)
μ_3_	1.51 (1.41–1.82)	29.4 ^*^ (21.9–35.4)^*^	0.00404 (0.00294–0.00556)	0.00277 (0.00200–0.00385)
***B. CEREUS***				
μ_1_	0.0218 (0.0156–0.0287)	0.0271 (0.0217–0.0343)	0.112 (0.0794–0.165)	0.212 (0.104–0.450)
μ_2_	2.17 (2.01–2.25)	178^*^(142–223)^*^	0.941 (0.645–1.27)	0.591 (0.381–0.918)
μ_3_	40.0 (27.3–72.0)	404^*^ (155–462)^*^	1.37 (0.801–2.32)	0.981 (0.57–1.67)

*Antibacterial potencies for P4LC, Pep-4, Pep-4/LVFX and LVFX expressed in terms of EC_50_ values (μM) and corresponding 95% confidence interval ranges. Ionic strengths of assay media correspond to: μ_1_ = 26 mM, μ_2_ = 111 mM, and μ_3_ = 167 mM*.

* *Estimated values are given for Pep-4 under conditions where it failed to achieve sufficient killing (within the tested peptide concentration range) to define the lower survival boundary for fitting to Equation 3*.

### DiSC (3)5 membrane depolarization assay

Enumerated frozen aliquots of *E. coli* and *B. cereus* cells were thawed and washed 3x with buffer (10 mM NaPO_4_, pH 7.4). The washed cells were diluted in 10 mM NaPO_4_ buffer containing 10 ug/mL DiSC(3)5 to afford a concentration of 4 × 10^7^ CFU/mL. Bacterial suspension was added to the wells of a black polypropylene microtiter plate (Greiner Bio-One) in 100 μL aliquots, and fluorescence was monitored (rt, with lid on) using a SpectraMax Gemini EM plate-reading fluorometer (622 nm_ex_/ 670 nm_em_) until fluorescence quenching stabilized and maximal DiSC(3)5 uptake was achieved. Aliquots (100 μL) of P4LC, Pep-4, LVFX, or Pep-4/LVFX were added to the bacteria and changes in fluorescence were monitored for 30 min with the lid off. The final concentrations of each assessed compound in the wells were 33 μM (P4LC and Pep-4), 126 μM (Pep-4/LVFX), and 93 μM (LVFX), while final bacterial concentrations were 2 × 10^7^ CFU/mL. Assays were performed in triplicate for each compound assessed. Depolarization assays based on DiSC(3)5 are extremely time-sensitive; therefore, triplicate sets for each compound were prepared simultaneously using a multichannel pipettor. Prior experiments verified that each tested compound was able to exert full killing under the assay conditions. A negative control containing only bacteria and DiSC(3)5 was used to establish background fluorescence, while maximal membrane depolarization was established using melittin (33 μM), a known pore-forming CAMP (Lee et al., 2013b); these controls provided references for establishing relative effectiveness of P4LC, Pep-4, LVFX, and Pep-4/LVFX. The fluorescence kinetics data sets were normalized by setting emitted fluorescence to 0 at *t* = 0 s and presenting fluorescence intensity values as a fraction of maximal fluorescence observed for melittin.

### SYTOX green membrane perturbation assay

Enumerated frozen aliquots of *E. coli* and *B. cereus* cells were thawed and washed 3x with buffer (10 mM NaPO_4_, pH 7.4). The washed cells were diluted to a concentration of 4 × 10^7^ CFU/mL in 10 mM NaPO_4_ (pH 7.4) buffer containing 5 μM SYTOX Green, and the resulting suspension then stored in the dark for ~20 min. The bacterial suspension was then distributed in 100 μL aliquots to wells of a black polypropylene microtiter plate (Greiner Bio-One). Aliquots (100 μL) of buffer containing P4LC, Pep-4, LVFX, or Pep-4/LVFX were then added to wells containing cell suspension and gently mixed. The fluorescence of each well was immediately monitored (without the lid) using a SpectraMax Gemini EM plate-reading fluorometer (485/520 nm_em_). As SYTOX Green assays can be time-sensitive, assays were prepared in triplicate using a multichannel pipettor. The reported SYTOX Green data reflects observations from biological triplicates. Antibacterial assays confirmed that each compound was able to exert full killing under these conditions and at the concentrations used in the DiSC(3) 5 assay (33 μM P4LC, 33 μM Pep-4, 126 μM Pep-4/LVFX, and 93 μM LVFX). A negative control containing only bacteria and SYTOX Green was used to determine background fluorescence, and maximal perturbation of the bacterial cell membranes was established using melittin. The fluorescence kinetics data sets were normalized by setting emitted fluorescence to 0 at *t* = 0 s and presenting fluorescence intensity values as a fraction of maximal fluorescence observed for melittin.

### Statistical analysis

An F test was used for determining whether log (EC_50_) values generated for the assessed compounds were significantly different from each other (computed using GraphPad Prism). For membrane depolarization and perturbation experiments, fluorescence data was analyzed by comparing best-fit curves (GraphPad Prism). Fluorescence kinetic curves were fit to a polynomial regression model (equation 4) to allow for statistical comparison of the fluorescence data *via* an F test, with all four best-fit parameters used as criteria.

(4) $$\text{RFU}={\text{A}}_{0}+{\text{A}}_{1}(\text{t})+{\text{A}}_{2}{\left(\text{t}\right)}^{2}+{\text{A}}_{3}{\left(\text{t}\right)}^{3}+{\text{A}}_{4}{\left(\text{t}\right)}^{4}$$

### Distribution coefficient

The relative hydrophobicities of P4LC, Pep-4, and LVFX were established using a biphasic system consisting of immiscible 1-octanol and 10 mM NaPO_4_ (pH 7.4) to determine their respective distribution coefficients (D_7.4_), with D_7.4_ corresponding to the concentration ratio of each compound between the octanol and aqueous phases (Brillault et al., 2010; Cheng et al., 2012; Chyan et al., 2014). In these experiments, P4LC, Pep-4 or free LVFX dissolved in 100 μL of 10 mM NaPO_4_ (pH 7.4) buffer was combined with 100 μL of 1-Octanol in a 1.5 mL low binding polypropylene microcentrifuge tube (Corning). Each tube was vortexed for 10 min and the contained solvent allowed to settle. Once the aqueous and octanol layers had reestablished and fully separated, 25 μL aliquots were removed from the aqueous phase using a micropipette, and the peptide or conjugate concentration determined using an absorbance-based BCA kit (Wrolstad et al., 2004). Aqueous phase Pep-4 and P4LC concentrations were determined *via* interpolation based on standard curves that had been generated using two-fold serially diluted peptide or conjugate dissolved in 10 mM NaPO_4_(pH 7.4). The maximal concentrations of Pep-4 and P4LC in the calibration curves were also established using amino acid analysis (UC Davis Genome Center Proteomics Core Facility). The results from amino acid analysis were in agreement with those obtained using the BCA kit. The concentration of LVFX in the aqueous layer was interpolated from calibration curves established using absorbance at 298 nm (LVFX absorbs strongly at this wavelength). Indolicidin and melittin were used as reference peptides in these studies. The sequence of indolicidin suggests that it is highly hydrophobic, while that of melittin indicates that it should be moderately hydrophobic. Computational verification of the hydrophobicity of these reference peptides was performed using the Hopp and Woods scale (Hopp and Woods, 1981). The D_7.4_ values generated for P4LC, Pep-4, LVFX, indolicidin, and melittin were calculated (equation 5) based on their respective concentrations in the aqueous phase (*[peptide]_PO4buffer_*) of the water/octanol biphasic system and their concentrations in the initial stock solution (*[peptide]_total_*). Values are averaged from six replicates.

(5) $$\begin{array}{l} \log{\text{D}}_{7.4}=\log\left(\begin{array}{l} ({\left[\text{peptide}\right]}_{\text{total}}-{\left[\text{peptide}\right]}_{\text{PO4buffer}})/\\ \left({\left[\text{peptide}\right]}_{\text{PO4buffer}}\right) \end{array}\right)\\ \;=\log\;({\left[\text{peptide}\right]}_{\text{octanol}}/{\left[\text{peptide}\right]}_{\text{PO4buffer}}) \end{array}$$

## Results

### Synthesis and purification of Pep-4-LVFX conjugate

**Figure 1:**
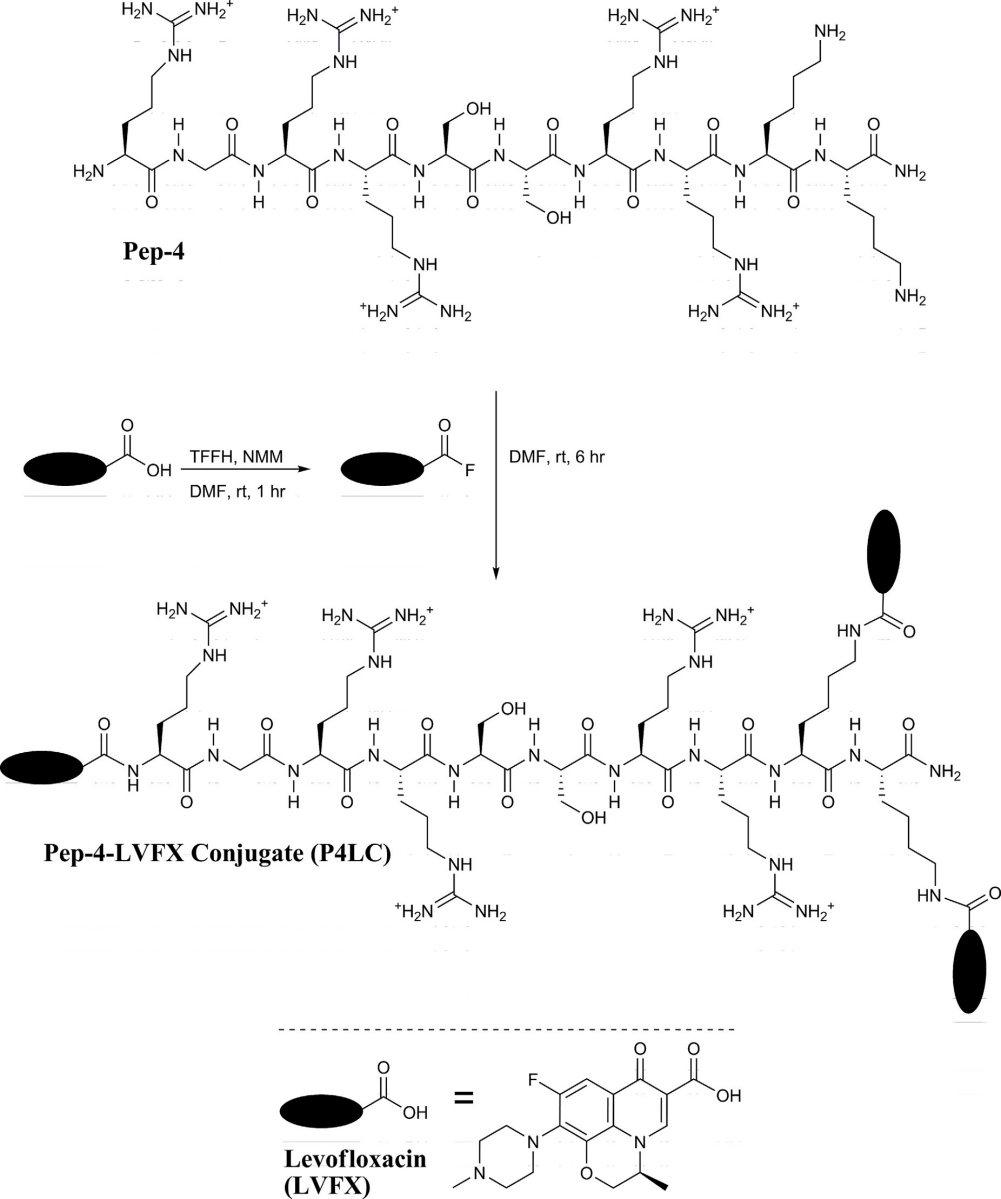
**Pep-4 was coupled to LVFX in a two-step, one-pot synthesis**. The carboxyl moiety of LVFX was preactivated using TFFH to afford the acyl fluoride. Pep-4 was then added to the activated LVFX reaction mixture to yield P4LC.

**Figure 2:**
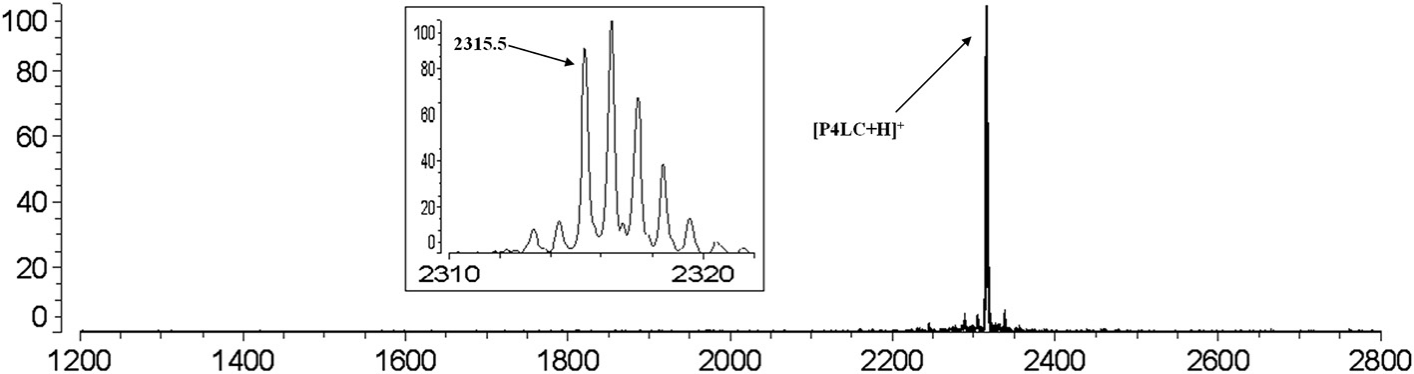
**MALDI-TOF mass spectrum of P4LC (m/z: 2315.5) obtained after purification indicates a 3:1 LVFX/Pep-4 loading stochiometry**. No peaks assignable to Pep-4 or intermediately acylated conjugate species were observed. The baseline isotopic distribution for P4LC is shown in the inset with labeling of the monoisotopic peak.

**Figure 3:**
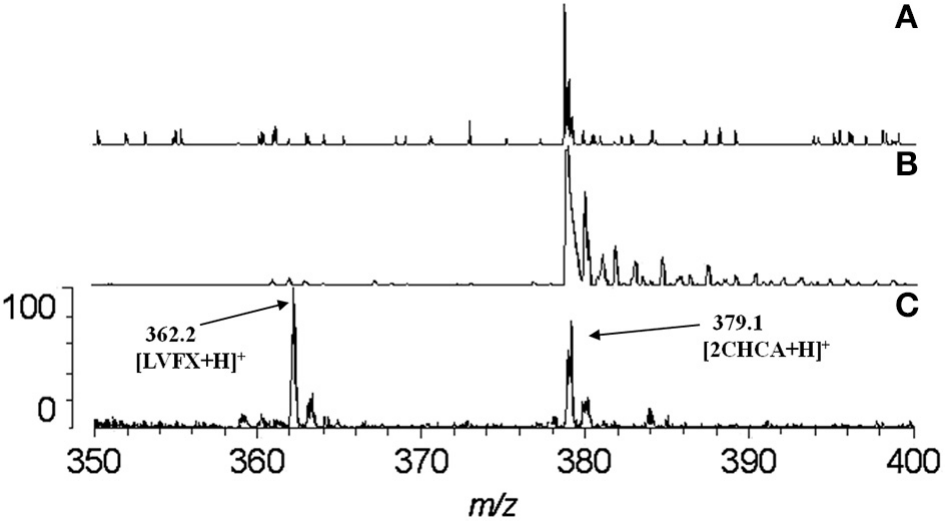
**Comparison of spectra at the region where LVFX m/z should appear**. The absence of any discernible peak near 362.2 in the spectrum for P4LC **(A)** indicates that no LVFX contamination was present in isolated conjugate product. Spectra for matrix alone **(B)** and LVFX alone **(C)** are shown for comparison. As evidenced in the spectra, matrix-related peaks did not interfere with detection of the peak assigned to LVFX.

The carboxyl moiety of LVFX allowed for conjugation to Pep-4 *via* direct acylation of N-terminal and lysine side chain amino groups on the peptide (Figure 1). LVFX was preactivated with TFFH to convert the carboxyl moiety to an acyl fluoride prior to introduction of Pep-4. Excess activated LVFX was captured using a glycine-Wang resin. The resulting particle suspension was filtered through a medium glass frit into cold ether in order to remove the resin and precipitate the desired conjugate. The precipitated product was dissolved in 10 mM NaCl and dialyzed against deionized water to remove water soluble, low molecular weight impurities and byproducts. This approach was employed because removal of intermediates and byproducts by chromatography and extraction proved challenging and resulted in significant loss of product. The isolated material was analyzed by MALDI-TOF mass spectrometry (Figure 2), which indicated the degree of drug loading to be 3:1 LVFX/peptide, with no free peptide detected. Moreover, no intermediate conjugate species were observed. Lower molecular weight regions of spectra from conjugate spectra were compared to the corresponding regions in reference spectra collected for free LVFX and matrix alone (Figure 3) in order to verify the absence of free drug and other low molecular weight impurities.

### Antibacterial effectiveness

**Figure 4:**
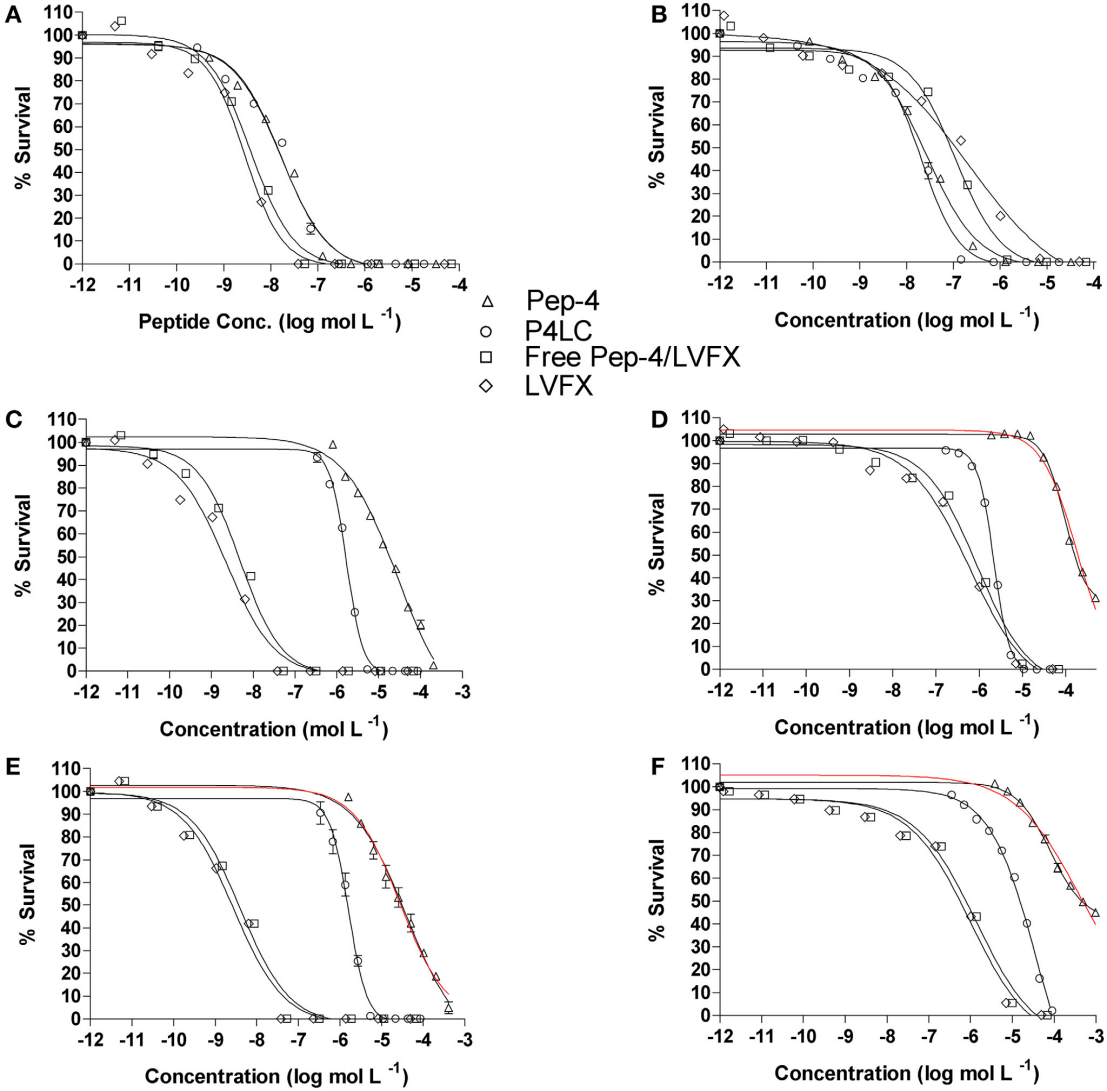
**Antibacterial effectiveness of P4LC, Pep-4, Pep-4/LVFX and LVFX against *E. coli* (A,C,E) and *B. cereus* (B,D,F)**. The top row **(A,B)** were generated for assays performed in low total ionic strength (26 mM), the middle row **(C,D)** for intermediate total ionic strength (111 mM), and the bottom row **(E,F)** for high total ionic strength (167 mM) conditions. Data were fit to equation 3, a standard variable slope dose-response equation, in order to obtain EC_50_ values. Pep-4 failed to achieve sufficient killing (within the tested peptide concentration range) to define the lower survival boundary for fitting to Equation 3 under higher ionic strength conditions **(D–F)**, and it was necessary to set the lower boundary to 0. Curves based on the data constraints are shown in red.

The antibacterial potencies of P4LC, Pep-4, and LVFX, as well as unmodified Pep-4 assayed in combination with free LVFX (Pep-4/LVFX), were evaluated against the model gram-negative and gram-positive bacteria *E. coli* and *B. cereus*, respectively. Assays were conducted under three different ionic strength (μ) buffer conditions in order to assess the effect salt concentration had on antibacterial effectiveness. The lowest ionic strength buffer used was 10 mM NaPO_4_, pH 7.4, which corresponded to 26 mM total ionic strength. The intermediate ionic strength buffer consisted of 10 mM NaPO_4_ combined with cation-adjusted PBS in a one to one volume ratio, which afforded a 111 mM ionic strength buffer with a pH of 7.4. Undiluted cation-adjusted PBS (pH = 7.4), with an ionic strength of 167 mM, was used as the high ionic strength buffer. Bacterial survival results were plotted against the log of the concentration of conjugate, peptide, or free drug. EC_50_ values for each assessed compound were calculated by fitting the survival data to equation 3, which models typical sigmoidal dose-response behavior using a variable Hill slope. An F test was used to assess statistical significance, with the alpha level set to 0.05. EC_50_ values and their respective 95% confidence intervals are shown in Table 1, and generated survival curves are shown in Figure 4.

In low ionic strength conditions (μ = 26 mM), P4LC demonstrated similar antibacterial activity to Pep-4 against both bacteria. Against *E. coli*, the conjugate demonstrated an EC_50_ of 0.0167 μM, which was not found to be significantly different than that of the free peptide, which displayed an EC_50_ value of 0.0168 μM (*p* = 0.91). Against *B. cereus*, P4LC demonstrated an EC_50_ value of 0.0218 μM, while Pep-4 displayed an EC_50_ of 0.0271 μM, values which were also determined not to be significantly different (*p* = 0.21). The combination of free LVFX and Pep-4 (Pep-4/LVFX) demonstrated an EC_50_ of 0.00375 μM against *E. coli*, while that of free LVFX was 0.00281 μM, values which were not significantly different from each other (*p* = 0.20). Against *B. cereus*, Pep-4/LVFX had an EC_50_ of 0.112 μM and free LVFX an EC_50_ of 0.212 μM, values which were approximately five-fold and ten-fold greater, respectively, than those determined for P4LC and Pep-4. The EC_50_ value of Pep-4/LVFX was not significantly different than that of free LVFX alone (*p* = 0.36) against *B. cereus*.

P4LC demonstrated EC_50_ values of 1.71 μM and 2.17 μM against *E. coli* and *B. cereus*, respectively, in intermediate ionic strength conditions (μ = 111 mM). These values are approximately 17-fold and 82-fold lower than those of Pep-4, 28.3 μM (*E. coli*) and 178 μM (*B. cereus*), under the same conditions. Against *B. cereus*, Pep-4 displayed antibacterial activity only at high peptide concentrations, killing a maximum of 69% of bacteria at the highest assessed peptide concentration (491 μM). In order to fit the data to Equation 3 and estimate the potency of Pep-4, it was necessary to set the lower boundary to 0%, a constraint not typically imposed. Therefore, the EC_50_ value given for Pep-4 against *B. cereus* under these conditions represents an estimated value. P4LC, LVFX, and Pep-4/LVFX provided sufficient data points in the lower survival ranges to fit the data by setting the lower survival boundary to ≥0%. Overall, the potencies of P4LC and Pep-4 decreased significantly compared to their respective performances against both bacteria under low salt conditions. Meanwhile, the potencies of Pep-4/LVFX (0.00487 μM) and free LVFX (0.00223 μM) against *E. coli* were not significantly affected by the increased ionic strength. However, in the case of *B. cereus*, the EC_50_ values for Pep-4/LVFX (0.941 μM) and free LVFX (0.591 μM) were significantly higher than those observed under low ionic strength conditions. The potencies of Pep-4/LVFX and LVFX were not found to be significantly different from each other for either *E. coli* (*p* = 0.071) or *B. cereus* (*p* = 0.11) under these conditions.

Under high ionic strength conditions (μ = 167 mM), the conjugate demonstrated EC_50_ values of 1.51 μM and 40.0 μM against *E. coli* and *B. cereus*, respectively. By comparison, the EC_50_ values achieved under high salt conditions with free peptide were 29.4 μM against *E. coli* and 404 μM against *B. cereus*. Thus, as was the case in the intermediate ionic strength environment, P4LC proved significantly more effective than Pep-4 (approximately 20-fold and 10-fold more potent against *E. coli* and *B. cereus*, respectively). Pep-4 failed to exert full killing activity under high ionic strengths within the range of concentrations evaluated, killing a maximum of 90% of *E. coli* (highest tested peptide concentration was 410 μM) and 56% of B cereus (highest peptide concentration tested was 983 μM). Therefore, it was necessary to set the lower survival boundary to 0% against both bacteria for Pep-4. The conjugate's EC_50_ against *E. coli* did not change significantly as a result of the ionic strength increase from 111 to 167 mM, while its effectiveness against *B. cereus* decreased by approximately an order of magnitude as a result of the change in environment. This trend was similar to that observed with Pep-4, where the peptide exhibited a substantial increase in its EC_50_ value against *B*. cereus, though not against *E. coli*, as a result of the assay ionic strength increase from 111 to 167 mM. Under high ionic strength conditions, the Pep-4/LVFX combination demonstrated an EC_50_ of 0.00404 μM against *E. coli* and free LVFX exhibited an EC_50_ of 0.00277 μM. The values were not found to be significantly different from each other (*p* = 0.51). Moreover, their high salt EC_50_ values were not significantly different from the EC_50_ values that they demonstrated under intermediate ionic strength conditions. Meanwhile, Pep-4/LVFX exhibited an EC_50_ of 1.37 μM against *B. cereus*, and free LVFX showed an EC_50_ of 0.981 μM, values that were determined to not be significantly different from each other or the corresponding values observed under intermediate ionic strength conditions.

### Membrane disruption

CAMPs have been shown to directly perturb bacterial membrane integrity, with the degree of inflicted disruption varying depending on both the peptide and the bacteria being targeted. CAMP-induced membrane disruption can range from the formation of minor transient gaps that result in depolarization of the membrane to the formation of larger perturbations and pores (Kaplan et al., 2011). The degree of disruption in bacterial cell membranes can be probed using fluorometric reporter molecules such as DiSC(3)5 (for depolarization) (Síp et al., 1990; Zhu and Shin, 2009) and SYTOX Green (for more substantial membrane disruption) (Luque-Ortega et al., 2008).

**Figure 5:**
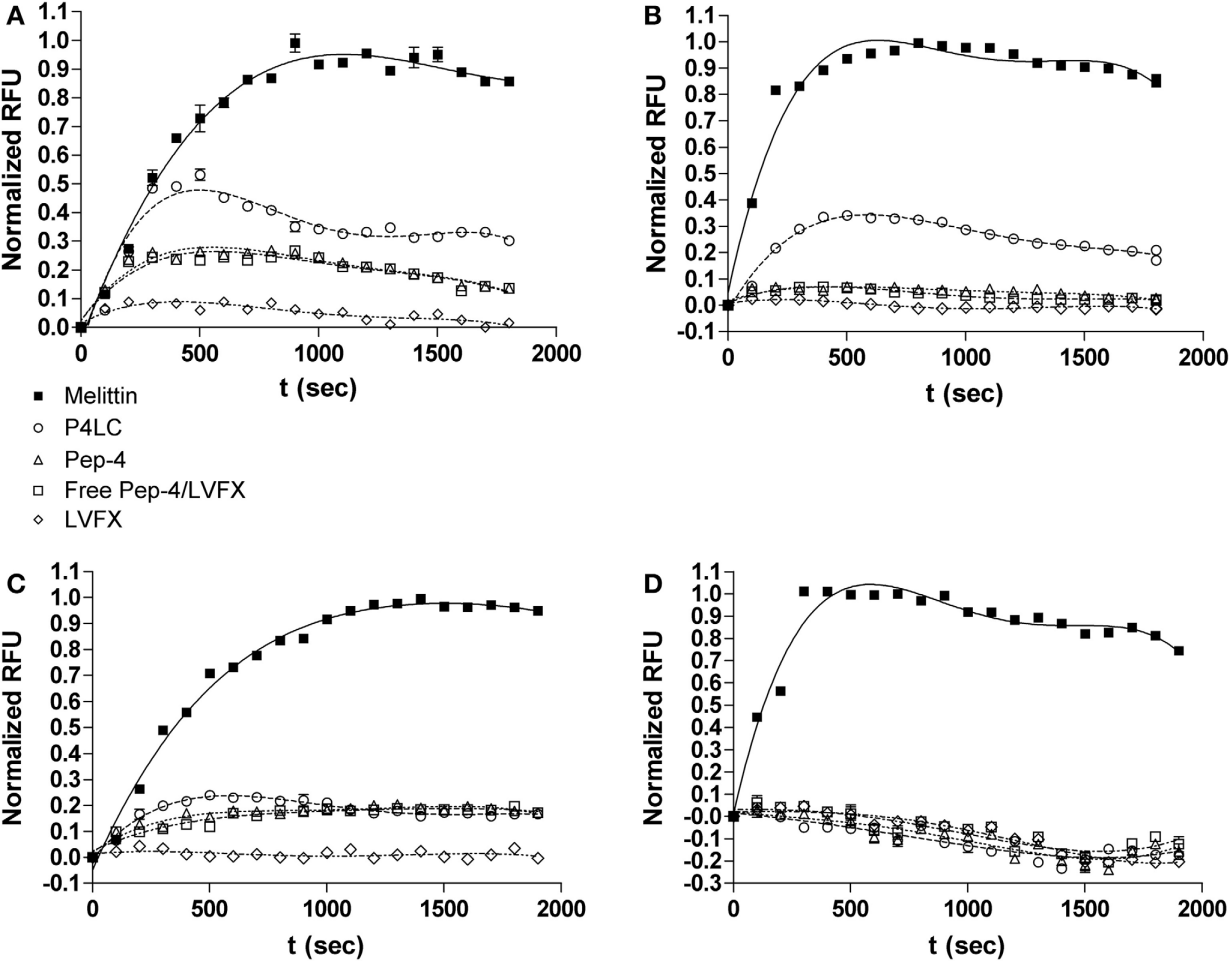
**Depolarization (top row) and perturbation (bottom row) of *E. coli* membranes (A,C) and *B. cereus* membranes (B,D) induced by P4LC, Pep-4, Pep-4/LVFX, LVFX and melittin**. Fluorescence values are presented as a percentage of the maximal fluorescence signal intensities observed for the pore-forming CAMP, melittin.

The ability of P4LC, Pep-4, LVFX, and Pep-4/LVFX to depolarize bacterial membranes was examined using the carbocyanin dye DiSC(3)5. When exposed to live cells, DiSC(3)5 concentrates in hyperpolarized membranes (Síp et al., 1990; Kaplan et al., 2011), which results in quenching of its fluorescence (622/670 nm_em_). Dissipation of membrane potential gradients resulting from even minor transient disruptions in lipid bilayer integrity inflicted by antibacterial agents such as CAMPs results in the release of trapped DiSC(3)5, which can be detected by monitoring DiSC(3)5 fluorescence (Rathinakumar et al., 2009; Zhu and Shin, 2009). In this study, fluorescence induction curves were generated for conjugate, peptide, or drug in the presence of *E. coli* and *B. cereus* cells that had been charged with DiSC(3)5. The data sets for P4LC, Pep-4, LVFX, and Pep-4/LVFX were compared to that obtained for melittin (Lee et al., 2013b), a known pore-forming CAMP that was used as a positive control to establish maximal depolarization. Data for DiSC(3)5 release was fit to a polynomial regression model (equation 4, see Materials and Methods) and these data were then subjected to an F test, with the alpha level set to 0.01, in order to allow comparison of curves and establish statistical significance of any observed similarities or differences. Fitted curves are shown in Figures 5A,B.

Addition of P4LC to wells containing *E. coli* and *B. cereus* led to a rapid increase in fluorescence in both cases. Compared to the results obtained for melittin, P4LC was able to cause 53.6 and 33.5% mean maximal fluorescence against *E. coli* and *B. cereus*, respectively. In contrast, Pep-4 caused 26.6% maximal fluorescence against *E. coli* and 7% against *B. cereus*. Similarly, Pep-4/LVFX induced 25% maximal fluorescence against *E. coli* and 7.4% against *B. cereus*. Finally, free LVFX was only able to cause 8.4% and 2.5% of maximal fluorescence against *E. coli* and *B. cereus*, respectively. The best-fit curves generated for P4LC and free LVFX were found to be significantly different from those of Pep-4 and Pep-4/LVFX, as well as from each other. Meanwhile, the curves generated for Pep-4 and for Pep-4/LVFX were found to not be significantly different from each other (*p* = 0.32 for *E. coli*; *p* = 0.071 for *B. cereus*).

The membrane impermeable reporter SYTOX green was used as a probe to gain further insight into the effects of P4LC, Pep-4, LVFX, and Pep-4/LVFX on *E. coli* and *B. cereus* cell membrane integrity. SYTOX Green has been shown to bind DNA, which results in a >500-fold increase in fluorescence intensity. The DNA found in intact bacteria is inaccessible to SYTOX Green binding, with fluorescence occurring only after the integrity of the bacterial membrane has been sufficiently compromised (Roth et al., 1997). Whereas DiSC(3)5 requires only minor and transient disruption in membrane integrity for its release from membranes, SYTOX Green requires more substantial perturbation to enter the bacterial cytoplasm and bind DNA. Accordingly, incubation of bacteria with CAMPs that perturb membrane integrity sufficiently for SYTOX green to enter the cytoplasm results in an increase in fluorescence (Lee et al., 2013b). As with the membrane depolarization studies described above, fluorescence curves were generated for P4LC, Pep-4, LVFX, and Pep-4/LVFX, and the data sets compared to those generated for melittin, which was used as the positive reference to establish maximal SYTOX Green fluorescence. Fluorescence induction curves were fit to equation 4 for statistical assessment (Figures 5C,D).

Addition of P4LC to wells containing *E. coli* led to a moderate increase in fluorescence; however, no noticeable increase in fluorescence was noted for *B. cereus*. In the case of *E. coli*, incubation with P4LC resulted in 21.4% mean maximal fluorescence relative to the results obtained for melittin. Pep-4 afforded 19.1% maximal fluorescence against *E. coli* and, similar to P4LC, yielded no noticeable fluorescence increase with *B. cereus*. The best-fit curve for P4LC when incubated with *E. coli* was found to be significantly different than that of Pep-4 (*p* < 0.0001), while the curves for Pep-4 and P4LC against *B. cereus* were not found to be significantly different (*p* = 0.44). Similar to P4LC and Pep-4, Pep-4/LVFX provided approximately one fifth of the maximal fluorescence increase when incubated with *E. coli* and no noticeable increase with *B. cereus*. The best-fit curves for Pep-4/LVFX were found to be significantly different than those of P4LC (*p* < 0.0001) for both bacteria. However, the best-fit curves for Pep-4/LVFX were determined to not be significantly different from those of Pep-4 (*p* = 0.012 for *E. coli*; *p* = 0.062 for *B. cereus*). No increase in fluorescence was noted when either bacterium was incubated with LVFX.

### Distribution coefficient

**Figure 6:**
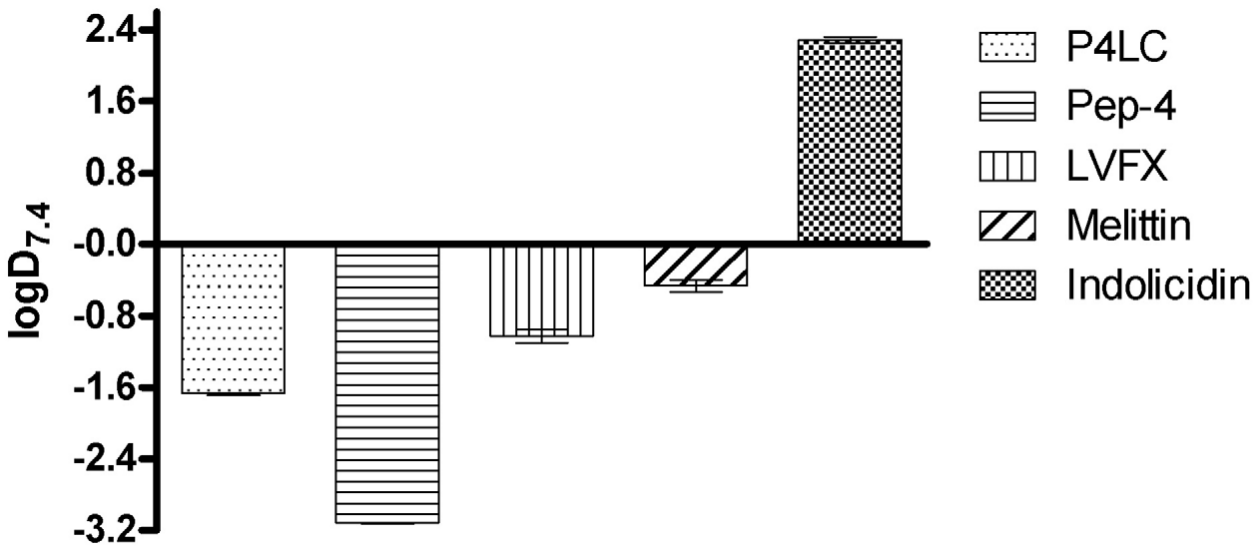
**Experimentally determined logD_7.4_ values for P4LC, Pep-4, LVFX, melittin, and indolicidin**.

Hydrophobic moment is known to be a crucial factor influencing CAMP membrane dynamics and overall antibacterial activity (Liu and Deber, 1998; Stark et al., 2002; Chen et al., 2007; Yin et al., 2012; Lee et al., 2013a). In order to gain insight into the relative hydrophobicities of P4LC, Pep-4, and LVFX, their partitioning between aqueous 10 mM NaPO_4_ (pH 7.4) and 1-octanol, a non-polar solvent that is immiscible with water, was assessed. The concentration ratios of each compound between the two liquid phases were used to establish distribution coefficients (D_7.4_). Indolicidin and melittin were used as reference peptides in these studies. Indolicidin is a short tryptophan and proline-rich CAMP with a high overall hydrophobicity (Podorieszach and Huttunen-Hennelly, 2010), while melittin is a well-studied peptide with an intermediately hydrophobic sequence (Li et al., 2013). LogD_7.4_values for Pep-4, P4LC, LVFX, melittin, and indolicidin are shown in Figure 6. Based on the results of these partitioning studies, Pep-4 was found to be the least hydrophobic of the CAMPs tested, demonstrating a logD_7.4_ of −2.57. By comparison, the logD_7.4_ of P4LC was found to be −1.65 (D_7.4_ = 0.0210), approximately an order of magnitude greater than that of Pep-4. Meanwhile, LVFX demonstrated a logD_7.4_ value of −1.05 (D_7.4_ = 0.094), which was about 4.5-fold greater than P4LC in terms of D_7.4_. The reference peptide indolicidin was found to have a logD_7.4_ value of 2.30, which was substantially higher than that observed for any other compound tested. The logD_7.4_ value for melittin, which was predicted to exhibit intermediate hydrophobicity, was determined to be −0.46, approximately an order of magnitude greater than that of the conjugate.

## Discussion

These studies have focused on the antibacterial performance of a peptide conjugate comprised of the fluoroquinolone LVFX covalently affixed to Pep-4, a ten-residue CAMP based on the c-terminal sequence of hBD-3. The conjugate, P4LC, was synthesized *via* acylation of primary amino groups in Pep-4 using the carboxyl moiety of LVFX. Our findings reveal that attachment of LVFX to Pep-4 yields a conjugate with significantly enhanced potency relative to the scaffold peptide in physiologically relevant ionic strength conditions. The results observed for unmodified Pep-4 coadministered with free LVFX suggests that the conjugate's antibacterial potency is not due to extracellular release of free drug.

The antibacterial effectiveness of P4LC, Pep-4, LVFX, and free peptide in combination with free drug (Pep-4/LVFX) were assessed against the model gram-negative and gram-positive bacteria, *E. coli* and *B. cereus*, respectively. Assays were conducted in various ionic strength environments, including physiologically relevant conditions, in order to evaluate the effect that salt concentration had on their antibacterial potencies. While P4LC and Pep-4 did not demonstrate significantly different EC_50_ values against either bacterium in the lowest ionic strength conditions tested (μ = 26 mM), the conjugate was found to be substantially more effective than the free peptide at higher ionic strengths. At an ionic strength of 111 mM, P4LC was found to be 17-fold more effective than Pep-4 against *E. coli* and 82-fold more effective against *B. cereus*. In the highest salt concentrations tested (μ = 167 mM), P4LC was 20-fold more effective than free peptide against *E. coli* and 10-fold more effective against *B. cereus*. Meanwhile, EC_50_ values determined for Pep-4/LVFX were significantly lower than those of the conjugate in all conditions, with the exception being the lowest ionic strength environment, where the conjugate was significantly more effective against *B. cereus*. These findings suggest that the enhanced activity of the conjugate relative to the free peptide is likely not due to the activity of extracellular LVFX release. Our findings also suggest that there was no synergistic enhancement of LVFX activity conferred by coadministration of unmodified Pep-4 with free LVFX, as EC50 values for Pep-4/LVFX were in most cases not found to be significantly lower than those of LVFX.

In order to gain insights into the antibacterial mechanisms of P4LC and Pep-4, experiments were performed to investigate their interactions with bacterial membranes. The fluorescent potentiometric probe DiSC(3)5 was used to assess the extent to which P4LC, Pep-4, LVFX, and Pep-4/LVFX were able to induce bacterial membrane depolarization. Compared to melittin, a known pore-forming CAMP, P4LC induced moderate levels of depolarization against both *E. coli* and *B. cereus*. Whereas, the depolarization affected by Pep-4 in *E. coli* and *B. cereus* was found be approximately a half and a third, respectively, of that caused by the conjugate. Similar to Pep-4, the Pep-4/LVFX combination afforded minimal depolarization, with fluorescence curves found not to be significantly different than those achieved using the free peptide alone. Meanwhile, LVFX induced almost no detectable depolarization. These results suggest that the covalently bound fluoroquinolone enhanced the peptide's ability to depolarize the membranes of both *E. coli* and *B. cereus* relative to the free peptide.

The membrane impermeable DNA-binding dye SYTOX Green was used to further probe the interactions of P4LC and Pep-4 with bacterial membranes. In the case of *E. coli*, P4LC and Pep-4 were observed to induce a moderate increase in fluorescence, equivalent to approximately one fifth of that observed for the positive reference melittin. Yet, the conjugate induced a more rapid increase in fluorescence than did the free peptide, a difference that was verified through statistical analysis of regression curves fit to the observed fluorescence kinetics data sets. In the case of *B. cereus*, almost no detectable fluorescence increase was observed for either P4LC or Pep-4. Meanwhile, the data sets acquired for Pep-4/LVFX against both bacteria more closely resembled those of Pep-4 than those of P4LC, an observation that was statistically verified. Treatment with free LVFX was not found to produce an increase in fluorescence against either bacterium. Taken together, these results suggest that P4LC and Pep-4 both induce a moderate degree of membrane perturbation against *E. coli*, but little, if any, perturbation of *B. cereus* membranes. However, this data also suggests that differences may exist in the membrane disruption mechanisms employed by P4LC and Pep-4 against *E. coli*.

It has been shown that alteration of peptide hydrophobicity can influence CAMP interaction with membranes (Dathe and Wieprecht, 1999; Podorieszach and Huttunen-Hennelly, 2010; Lee et al., 2013a). Introduction of the moderately lipophilic LVFX would be expected to effectively increase the hydrophobic character of the conjugate relative to the unmodified parent peptide. Using a biphasic 1-octanol/phosphate partitioning system, we found that the conjugate was approximately one order of magnitude more hydrophobic than Pep-4 based on their respective dissociation constants, D_7.4_ (Pep-4 logD_7.4_= −2.57 vs. P4LC logD_7.4_= −1.65). This finding was consistent with the observation that the hydrophobicity of the free drug was approximately one and a half orders of magnitude greater than that of free Pep-4 (LVFX logD_7.4_= −1.05). The logD_7.4_ determined for LVFX that is reported here is in agreement with previously reported values (Brillault et al., 2010; Lemaire et al., 2011). Interestingly, it has been shown previously that increasing the hydrophobicity of ten-residue peptides with similar sequences to Pep-4 resulted in enhanced membrane interactions and reduced sensitivity to environmental salt concentration (Bai et al., 2009). The enhanced hydrophobicity conferred by the attached LVFX groups noted here is likely a factor contributing to the more substantial membrane interactions and increased salt resistance observed for the conjugate relative to the free peptide.

## Conclusion

In an effort to explore design parameters for engineering CAMP conjugates with enhanced properties, a novel antimicrobial peptide conjugate, P4LC, was generated by attaching multiple units of the fluoroquinolone antibiotic LVFX to Pep-4, a ten residue CAMP that presents multiple primary amino groups suitable for drug attachment via acylation. Our studies revealed that while P4LC displayed similar antibacterial effectiveness as the unmodified parent peptide Pep-4 under low salt conditions, it was substantially more potent under physiologically relevant high salt conditions. This finding is significant because most CAMPs have historically been found to lose antibacterial effectiveness in physiological ionic strength environments, a fact which has presented a challenge to the development of these peptides for therapeutic applications. Our studies also suggest that the conjugate's antibacterial potency is not due to extracellular release of free drug, as evidenced by the results observed for unmodified Pep-4 coadministerd with free LVFX. Partitioning studies revealed that P4LC was more hydrophobic than Pep-4, while depolarization studies indicated that the conjugate was able to disrupt membrane integrity to a greater degree than the free peptide. We propose that the conjugate's observed increased hydrophobicity plays a role both in its enhanced salt resistance and ability to depolarize bacterial membranes. However, the influence that the LVFX groups have on the performance of the conjugate may not solely be attributable to the increased hydrophobicity. In our prior studies with Pep-4, acetylation of its amino groups was found to decrease the effective positive charge character of the peptide, which resulted in a significant reduction in potency under low salt conditions. In contrast, acylation of Pep-4 with LVFX did not result in a loss of potency under similar conditions. The superior performance of P4LC, relative to acetylated Pep-4, could reflect contributions from multiple factors. Unlike acetyl groups, LVFX contains protonatable nitrogens that could contribute to the overall cationic character of the conjugate. Additionally, the affixed LVFX groups may contribute some aspects of their inherent antimicrobial activity to that of the conjugate. Future efforts will focus on further investigating factors contributing to the antibacterial properties of P4LC and related conjugates. Parameters contributing to more effectively capturing the inherent antibacterial potency of LVFX will be of particular interest. Insights gained from studying the performance and physicochemical properties of P4LC will provide valuable information that can be used in the design of new conjugates incorporating different peptide scaffolds and a broader lexicon of auxiliary molecules, including antibiotics or chemotherapeutics.

### Conflict of interest statement

The authors declare that the research was conducted in the absence of any commercial or financial relationships that could be construed as a potential conflict of interest.
